# Characterisation and Antioxidant Activity of Crude Extract and Polyphenolic Rich Fractions from *C. incanus* Leaves

**DOI:** 10.3390/ijms17081344

**Published:** 2016-08-17

**Authors:** Antonella Gori, Francesco Ferrini, Maria Cristina Marzano, Massimiliano Tattini, Mauro Centritto, Maria Camilla Baratto, Rebecca Pogni, Cecilia Brunetti

**Affiliations:** 1 Department of Agrifood Production and Environmental Sciences (DiSPAA), University of Florence, 50019 Sesto Fiorentino (Florence), Italy; antonella.gori@unifi.it (A.G.); francesco.ferrini@unifi.it (F.F.); mariacristina.marzano@unifi.it (M.C.M.); 2 Trees and Timber Institute (IVALSA), The National Research Council of Italy (CNR), 50019 Sesto Fiorentino (Florence), Italy; mauro.centritto@cnr.it; 3 Institute for Plant Protection (IPSP), The National Research Council of Italy (CNR), 50019 Sesto Fiorentino (Florence), Italy; massimiliano.tattini@ipsp.cnr.it; 4 Department of Biotechnology, Chemistry and Pharmacy, University of Siena, 53100 Siena, Italy; mariacamilla.baratto@unisi.it (M.C.B.); rebecca.pogni@unisi.it (R.P.)

**Keywords:** polyphenolic enriched fractions, flavonols, LC–MS/MS (liquid chromatography–tandem Mass Spectrometry), DPPH radical-scavenging activity

## Abstract

*Cistus incanus* (Cistaceae) is a Mediterranean evergreen shrub. *Cistus incanus* herbal teas have been used as a general remedy in traditional medicine since ancient times. Recent studies on the antioxidant properties of its aqueous extracts have indicated polyphenols to be the most active compounds. However, a whole chemical characterisation of polyphenolic compounds in leaves of *Cistus incanus* (*C. incanus*) is still lacking. Moreover, limited data is available on the contribution of different polyphenolic compounds towards the total antioxidant capacity of its extracts. The purpose of this study was to characterise the major polyphenolic compounds present in a crude ethanolic leaf extract (CEE) of *C. incanus* and develop a method for their fractionation. Superoxide anion, hydroxyl and DPPH (1,1-diphenyl-2-picrylhydrazyl) radical scavenging assays were also performed to evaluate the antioxidant properties of the obtained fractions. Three different polyphenolic enriched extracts, namely EAC (Ethyl Acetate Fraction), AF1 and AF2 (Aqueos Fractions), were obtained from CEE. Our results indicated that the EAC, enriched in flavonols, exhibited a higher antiradical activity compared to the tannin enriched fractions (AF1 and AF2). These findings provide new perspectives for the use of the EAC as a source of antioxidant compounds with potential uses in pharmaceutical preparations.

## 1. Introduction

Plants inhabiting Mediterranean-type ecosystems are usually challenged by multiple stressors, particularly during the summer, when water deficiency co-occurs with high solar irradiance and high temperatures. These environmental constraints induce severe photo-oxidative stress in Mediterranean plants [1,2], resulting in the formation of many reactive oxygen species (ROS). Reactive oxygen species include both radicals, such as superoxide anion and hydroxyl radicals, and non-free radicals, such as hydrogen peroxide and singlet oxygen. Within the plant cell, a first line of defense against reactive oxygen species is constituted by antioxidant enzymes [3]. In particular, superoxide dismutase (SOD) detoxifies superoxide anion (O_2_**^·^** ^−^) by converting two O_2_**^·^** ^−^ into H_2_O_2_ and O_2_ [4]. Furthermore, in the presence of O_2_**^·^** ^−^ and transition metal ions, H_2_O_2_ can generate hydroxyl radical (^•^OH) via the superoxide-driven Fenton reaction [3]. The ^•^OH is highly reactive, causing damage to DNA and lipid peroxidation [5]. Alterations in cellular ROS/REDOX homeostasis induce the activation of additional antioxidant defense systems constituted by secondary metabolites [2]. In particular, polyphenols have been widely reported to protect plants against oxidative stress [6], neutralising ROS, chelating transition metals and reducing lipid peroxidation [7,8,9].

New evidence suggests that polyphenols also have “indirect” antioxidant effects both in plants and humans [10]. The mechanisms by which polyphenols express these beneficial effects in vivo is not yet clear but it appears to involve their interaction with cellular signaling pathways [11,12]. In particular, polyphenols are thought to have the ability to interact with a wide range of protein kinases that supersede key steps of cell growth and differentiation [13]. Interestingly, the same structural features conferring antioxidant activity to polyphenols are also responsible for their ability to regulate these developmental processes [10]. Though such functions have not been conclusively proven in plant cells, they form the basis of the beneficial effects exerted by polyphenols in a wide range of diseases in animals, including their anti-cancer properties.

Mediterranean shrub species, such as *Cistus incanus*, are naturally rich in polyphenols and might represent a source of bioactive compounds for the development of novel drugs [14]. In traditional medicine, *C. incanus* herbal infusions have been used as anti-inflammatory agents in the treatment of various skin diseases [15,16]. Furthermore, *C. incanus* polyphenolic-rich extracts have been reported to possess antimycotic, antibacterial and antiviral properties [17,18,19,20,21]. Recently, aqueous extracts of the aerial parts of this plant have been demonstrated to exert intense antioxidant capacities that could be attributed to their high polyphenol content [22,23].

To the best of our knowledge, a complete chemical characterisation of the polyphenolic composition of the leaves of *C. incanus* has not yet been reported. Moreover, detailed antioxidant activities of different enriched fractions have not been investigated. Consequently, limited data is available on the contribution of the different polyphenolic compounds to the total free radical scavenging activity of leaves of this species. This study aims to characterise the major polyphenolic compounds contained in a crude ethanolic leaf extract (CEE) of *C. incanus*, and to develop an extraction protocol to obtain tannin and flavonol enriched fractions. Finally, scavenging activity against superoxide anion, hydroxyl and DPPH (1,1-diphenyl-2-picrylhydrazyl) radicals have been used to compare the antioxidative properties of CEE and its derived fractions. 

## 2. Results and Discussion

### 2.1. Qualitative Characterisation of Phenolic Compounds Present in Crude Extract of Cistus incanus (C. incanus) Leaves

In our study, HPLC–DAD-MS/MS was performed to assess the polyphenolic composition of a crude ethanolic extract (CEE) of *C. incanus* leaves. Individual polyphenols were identified on the basis of their fragmentation patterns as well as by comparison of their retention time and UV–VIS spectra with those of authentic standards.

**Figure 1:**
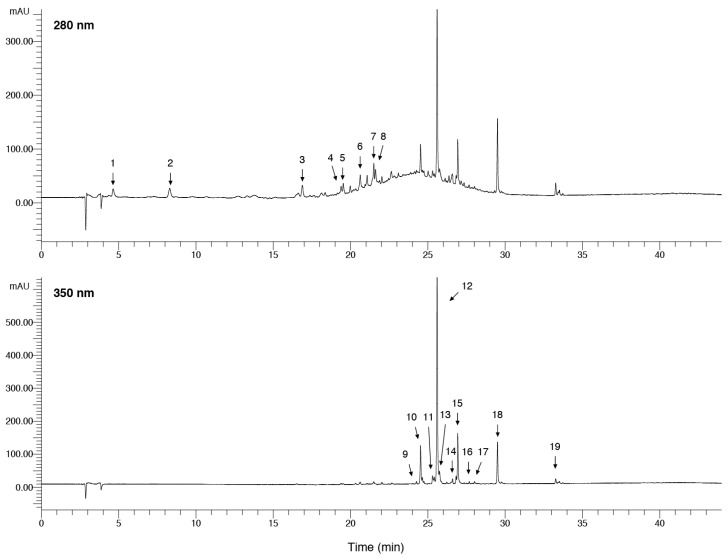
Chromatographic profile of crude ethanolic leaf extract (CEE) of leaves of *Cistus incanus* acquired by HPLC–DAD detected at the relative maxima of absorbance of proanthocyanins (280 nm) and flavonols (350 nm), respectively. Chromatographic conditions are given in the Materials and Methods section. For compound identification see Table 1.

Our analytical conditions allowed the separation of a large percentage of compounds, as shown in Figure 1.

The MS data obtained by liquid chromatography–tandem mass spectrometry (LC–MS/MS) of the most representative phenolics present in the CEE of *C. incanus* are listed in Table 1, identified with the numbers 1–19 according to their elution order. The compounds identified were classified in to three main classes: gallic acid derivatives (peaks 1, 2), condensed tannins (peaks 3–8), also known as proanthocyanidins, and flavonol glycosides (peaks 9–19). Peak 1 was identified as monogalloyl glucose (m/z at 331), with the main fragments at m/z 169 (gallic acid) and 125 (loss of CO_2_ from gallic acid). Peak 2 was identified as gallic acid (m/z 169) as previously reported by [22,24]. Condensed tannins, both monomeric, dimeric and polymeric proanthocyanidins have been already reported in *C. incanus* extracts [16].

Our chromatographic method was suitable for the determination of two dimeric (3, 6) and two monomeric proanthocyanidins (4, 5). In particular, as expected by the general scheme proposed by [25,26], the loss of a phloroglucinol unit (C_6_H_6_O_3_), as well as losses due to Retro-Diels-Alder (RDA) fission and interflavanoid cleavage, were the predominant fragmentation pathways of dimeric proanthocyanidins.

**Figure 2:**
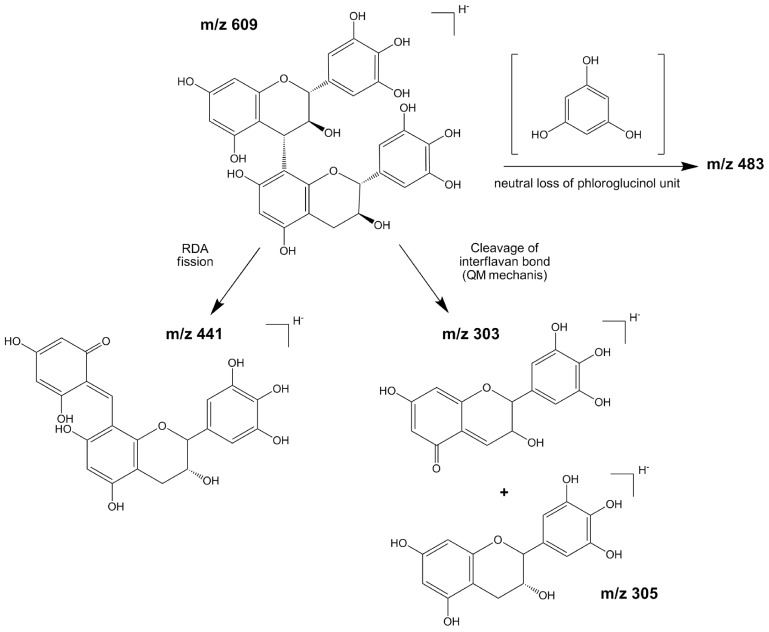
Hypothetical ESI(−)-MS/MS fragmentation pattern for Epigallocatechin dimer (peak 3, [M-H]^−^ at m/z 609). RDA = Retro-Diels Alder fission, QM = Quinone Methide cleavage mechanism.

On this basis, the fragmentation pattern of the epigallocatechin dimer (peak 3, [M-H]^−^ at 609) was consistent with an RDA fission of the heterocyclic ring resulting in the fragment ion at m/z 441 [23]. Furthermore, the fragments detected at m/z 303 (methylenic quinone) and m/z 305 (flavan-3-ol monomer) derived from an inter-flavanic bond cleavage, through the quinine methane (QM) cleavage mechanism, whereas the fragment ion at m/z 483 resulted from the loss of a phloroglucinol unit (Figure 2), [27]. According to [15], gallocatechin-(4α-8)-gallocatechin or the regio-isomer gallocatechin-(4α-6)-gallocatechin were strongly suggested as molecular structure for this dimeric proanthocyanidin.

At 20.6 min (peak 6) another dimeric proanthocyanidin was recorded. Its pseudomolecular ion peak [M-H]^−^ at m/z 593 suggested that this compound consisted of one (epi)gallocatechin and one (epi)catechin subunit [25]. MS/MS fragmentation of m/z 593 gave a fragment ion at m/z 425 from RDA rearrangement [28]. The sequential water elimination produced the ion at m/z 407 and the QM cleavage of the interflavonol bond produced a fragment ion at m/z 289. Finally, the ion at m/z 467 resulted from the loss of a C_6_H_6_O_3_^−^ fragment from the pseudomolecular ion. For this dimeric structure gallocatechin-(4α-8)-catechin or catechin-(4α-8)-gallocatechin is suggested according to [15].

Two monomeric gallocatechins were identified at 19.5 and 19.6 min (peaks 4 and 5). In particular, (−)-gallocatechin and its isomer (−)-epigallocatechin with [M-H]^−^ at 305 m/z were detected. Their molecular weight was confirmed by the presence of the ion at m/z 611 corresponding to [M + M-H]^−^. Further ions were detected at m/z 137, ([M-H-C_8_H_8_O_4_]^−^) resulting from retro RDA fission, and at m/z 125, corresponding to the loss of CO_2_ from gallic acid. In addition, both (+)catechin and (−)epicatechin (289 m/z) were found in the CEE (peaks 7 and 8) by comparison with fragmentation patterns of commercial standards.

Polymeric proanthocyanidins could not be resolved by reversed-phase HPLC as revealed by the unresolved hump between 22 and 28 m (Figure 1), as also previously reported by other authors [27,29].

**Figure 3:**
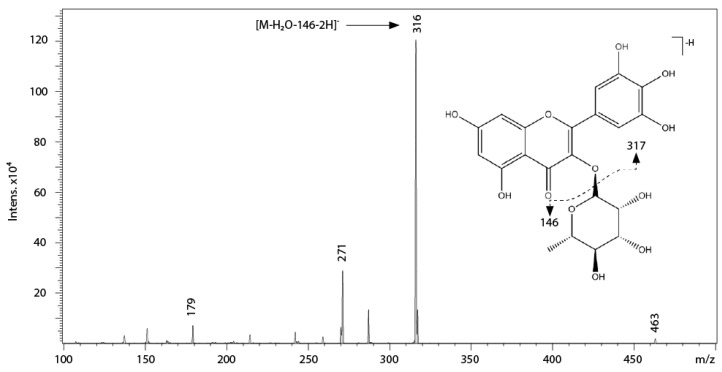
Structure, fragmentation and MS/MS spectrum of peak 12 (myricitrin). Solid arrow indicates the most abundant ion in myricitrin fragmentation; dashed arrow indicates the loss of rhamnose moiety.

Ten compounds were identified as flavonols. As occurred in other members of *Cistus* subgenus [28], myricetin-3-*O*-hexoside (10) and myricitrin (12) were present in the CEE. Fragmentations of the precursor ions at m/z 479 (10) and at m/z 463 (12, Figure 3) had a common fragment at m/z 316 [M-H_2_O-Hexose-2H]^−^, which could be attributed to myricetin [30,31,32,33]. The neutral loss of sugar units (losses of 162 for the hexose and 146 for the rhamnose moieties from compounds 10 and 12, respectively) and the product ion at m/z 271, typical of 3-*O*-monoglycosides [34], confirmed the presence of these compounds.

**Figure 4:**
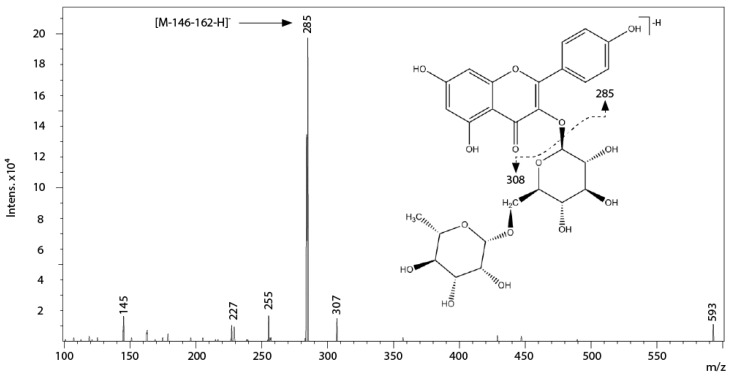
Structure, fragmentation and MS/MS spectrum of peak 18.

Peaks 13, 14 and 15 with precursor ions at m/z 609, 433 and 447 respectively, were identified as quercetin derivatives on the basis of the presence of their aglycone fragment (m/z 301). Particularly, peak 13 was positively identified as rutin, peak 14 as quercetin-3-*O*-pentoside, and peak 15 as quercitrin [22,24]. Peak 18 was identified as a kaempferol 3-*O*-rutinoside, on the basis of the pseudomolecular ion [M-H]^−^ at m/z 593 and the fragment at m/z 285 ([M-146-162-H]^−^), due to the loss of a glucosyl and a rhamnosyl moiety in an unique fragment (Figure 4). This fragmentation pattern is characteristic of flavonol rutinosides, in which the linkage 1–6 between rhamnose and glucose, that forms rutinose, allows for free rotation and a more accessible fragmentation than other disaccharides [35,36]. In accordance with [19], peak 19 was assigned as kaempferol-3-(3″,6″-dicoumaroyl)-glucose with a molecular ion at m/z 739 and a fragment at m/z 285.

Other flavonols have been tentatively identified as myricetin derivatives (peaks 9 and 11) and as quercetin derivatives (peaks 16 and 17) based on their retention times and their UV–VIS spectra, in the absence of conclusive mass-spectrometric data.

### 2.2. Antiradical Activity Evaluation of Different Extracts of Cistus incanus (C. incanus) Leaves

**Figure 5:**
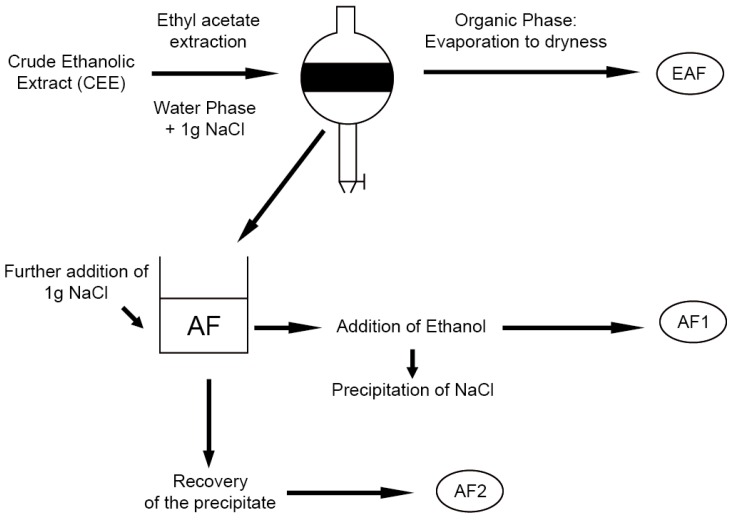
Scheme for fractionation of the CEE. EAF = Ethyl acetate Fraction, AF = Aqueous Fraction, AF1 = Aqueous Fraction 1, AF2 = Aqueous Fraction 2.

The CEE was partitioned following the protocol in Figure 5. The application of our partitioning process resulted in three different fractions enriched in distinct classes of polyphenols, one ethyl acetate flavonol enriched fraction (EAF) and two aqueous tannin enriched fractions (AF1 and AF2).

Compounds contained in the different extracts were identified and quantified by HPLC–DAD. The EAF was mainly composed of flavonol glycosides and oligomeric proanthocyanidins (monomers and dimers) whereas the two aqueous fractions contained only low and high polymeric proanthocyanidins (AF1 and AF2, respectively). These results are shown in Table 2.

The potential antioxidant activities of the different fractions were compared using three in vitro assays based on the scavenging of reactive oxygen species or stable free radicals: superoxide anion radical-scavenging, hydroxyl radical-scavenging and DPPH-scavenging assay (Figure S1 in supplementary material). Table 3 illustrates the IC_50_ values. IC_50_ denotes the concentration of the sample required to scavenge 50% of free radicals. These values were obtained from the regression equations, plotting extract concentrations against inhibition percentages of free radical formation in the different assays.

#### 2.2.1. Superoxide Anion Radical (O_2_ **^·^** ^−^) and Hydroxyl Radical Scavenging Activities

As shown in Table 3, the superoxide scavenging activity of different extracts of *C. incanus* leaves was found to occur in the following order: EAF >> CEE > AF1 and AF2. Our results indicate that lowest IC_50_ value is related to the highest concentration of flavonol compounds, as confirmed by the IC_50_ value of myricitrin standard. As already reported by Salaris et al. [37] polyphenols may act in two ways, by the direct scavenging of O_2_ **^·^** ^−^ and by the inhibition of xanthine oxidase enzyme, thus preventing the generation of this radical. In particular, Cos et al. [38] showed that catechin derivatives are superoxide scavengers without inhibitory activity on xanthine oxidase, whereas myricitin and quercetin derivatives display both activities. Furthermore, these flavonols have lower IC_50_ values for the reduction of superoxide level than for the inhibition of xanthine oxidase [39].

The highest antiradical scavenging activity of EAF was confirmed also by the hydroxyl radical scavenging assay (Table 3). Among the various extracts tested, this fraction displayed the lowest IC_50_, which is around half the values of the aqueous fractions (AF1 and AF2).

The ability of the EAF to quench hydroxyl radicals could be related to the capacity of some flavonols to form stable radicals. This mechanism has not been completely clarified; however, they could act as hydrogen donors, breaking radical chains through the formation of aroxyl radicals. The final products of these reactions are stable quinonic structures [40].

#### 2.2.2. 1,1-Diphenyl-2-picrylhydrazyl (DPPH) Radical Scavenging Activity

Results show that the highest DPPH radical scavenging activity was performed by EAF (IC_50_ = 0.92 ± 0.097), whereas the aqueous fractions had the highest IC_50_ values (11.78 ± 0.85 for AF1 and 10.92 ± 0.38 for AF2, respectively). The crude ethanolic extract exhibited an IC_50_ value of 2.99 ± 1.18, closer to EAF than to AFs (AF1 and AF2). Our results clearly indicate that the DPPH radical-scavenging activity was greatly influenced by the phenolic composition of the samples. In particular, flavonols (myricetin and quercetin derivatives) were dominant contributors to the DPPH radical scavenging activity of *C. incanus* extracts. Nevertheless, although high levels of proanthocyanidins were found in the aqueous extracts, these compounds did not seem to contribute significantly to the antiradical activity of the CEE measured by the DPPH method. Furthermore, no statistical difference was found between AF1 and AF2, suggesting that differences in the degree of polymerization of proanthocyanidins had relatively little effect on their overall quenching capacity.

#### 2.2.3. Structural Aspects of in Vitro Antiradical Activity of *C. incanus* Leaf Extracts

Our data shows a stronger antiradical capacity of EAF than AFs in all the tested assays. Furthermore, the antiradical capacity of *C. incanus* extracts is largely influenced by their polyphenolic composition. These results are in agreement with previous studies on other members of *Cistus* subgenus. For example, n-butanolic and ethyl acetate fractions of *C. laurifolius* displayed the highest flavonol content and also exerted the highest antioxidant activity in DPPH and FRAP (Ferric Reducing Antioxidant Potential) assays [41]. Tomas et al. [42] observed that the antioxidant capacities of *C. salvifolius* extracts in FRAP and TBARS (Thiobarbituric Acid Reactive Substances) assays increased considerably when these were concentrated in some specific flavonols. Numerous authors have investigated the antioxidant activity of polyphenols and several studies have been undertaken to establish the relationship between their structure and their radical-scavenging activity. The radical-scavenging activity of polyhenols depends upon the substitution pattern of their hydroxyl groups, i.e., on the availability of phenolic hydrogens and on the possibility of stabilization of the resulting phenoxyl radicals via hydrogen bonding or by electron delocalization [43]. In particular, the structural requirements considered to be essential for effective radical scavenging are: (i) the presence of a ortho-OH structure (catechol group in the B ring); (ii) a 2,3- double bond conjugated with the 4-oxo group. Moreover, compounds that contain multiple hydroxyl substitutions showed stronger antiperoxyl radical activities [44,45,46]. Among the compounds identified in *C. incanus* leaf extracts, myricitrin satisfies meets all of these criteria. In contrast, a flavan-3-ol such as catechin, which lacks of the 2,3- double bond and the 4-oxo function, is unable to support electron delocalization between the A- and the B-rings limiting its radical scavenging potential. This is supported by the comparison of IC_50_ values of myricitrin and epicatechin standards, since IC_50_ of myricitrin was approximately half the value of epicatechin in all the three assays (Table 3). Conversely, some galloylated catechins benefit from the contribution of esterification with gallic acid (3,4,5-trihydroxybenzoic acid), which compensates for the lack of electron delocalization with major electrondonating properties. This is the case of the (epi)gallocatechin dimer present in the EAF that could participate in the enhancement of its antioxidant activity. However, the presence of many hydroxyl groups in polymeric proanthocyanidins did not increase their scavenging capacity. As previously described by other authors [47,48,49], the chemical structure of polymeric proanthocyanidins may cause stereochemical hindrances, resulting in relatively high IC_50_ values of AF1 and AF2.

## 3. Materials and Methods

### 3.1. Plant Material and Extraction Procedure

Fully-expanded leaves from adult plants of *Cistus incanus* growing on seashore dunes in Southern Tuscany (42°46′ N, 10°53′ E) were harvested in July 2015. Plant material was rapidly frozen in liquid nitrogen and stored at −80 °C before proceeding with the analysis. 5 g of fresh plant tissue was ground in a mortar with liquid nitrogen. The obtained powder was extracted with 70% of aqueous ethanol acidified to pH 2.5 by HCOOH (50 mL × 5) and sonicated for 30 m. The solution was then partitioned with n-hexane (50 mL × 5) to completely remove lipophilic compounds, following the protocol previously reported by Romani et al. [50]. The ethanolic phase constituted the crude ethanolic extract (CEE). 125 mL of the CEE were then evaporated under vacuum (Rotavapor 144R, Buchi, Switzerland), re-dissolved in 250 mL of water and extracted five times with 50 mL ethyl acetate (*v*/*v*) (Figure 5). 1 g of NaCl was added to break down the emulsion and to accelerate the phase-separation process (“salting out” effect). The organic phase (ethyl acetate fraction, EAF) consisted mostly of flavonols, while the aqueous fraction (AF) contained tannins. Two more distinctive fractions (AF1 and AF2) were obtained by a successive precipitation through the addition of NaCl (1 g) to AF. This process was carried out to obtain the separation between low and high molecular weight polymeric proanthocyanidins following a modified protocol from Saucier et al. [51]. The precipitate formed was collected by filtration on glass filters (AF2), while the filtrate was added with ethanol to precipitate the salt and recover AF1. Finally, the CEE and AF1 were totally evaporated. All fractions were re-dissolved in 2.5 mL of water:ethanol, 80:20. An aliquot of each extract (300 µL) was diluted in 1.20 mL of methanol and acid water (pH 2 by HCOOH) 80:20 (*v*/*v*) and used for polyphenol analysis by HPLC–DAD and HPLC–MS.

### 3.2. Chemicals and Reagents 

The phenolic standards gallic acid, epicatechin, myricetin 3-*O*-rhamnoside, quercetin 3-*O*-rhamnoside, rutine and kaempferol 7-*O*-glucoside were obtained from Extrasynthese (Genay Cedex, France). FeSO_4_, hydrogen peroxide, sodium salicylate, xanthine, xanthine oxidase, nitro blue tetrazolium (NBT), ethylenediaminetetraacetic acid (EDTA), formic acid, ethanol, n-hexane, methanol and acetonitrile of HPLC purity were purchased from Sigma Aldrich (Milan, Italy). DPPH (2,2-diphenyl-1-picrylhydrazyl) was obtained from Merck (Darmstadt, Germany). Distilled water was purified in a milli-Q water purification system (Millipore Corporation, Bedford, MA, USA).

### 3.3. HPLC–DAD and LC–ESI-MS/MS Anlaysis of Phenolic Compounds

Identification of individual phenolics was carried out using their retention times and both UV–VIS, MS and MS/MS spectra. The LC–DAD-MS/MS system consisted of a Shimadzu LCMS-8030 quadrupole mass spectrometer (Kyoto, Japan) operated in the electrospray ionization (ESI) mode and a Shimadzu Nexera HPLC system (Kyoto, Japan) equipped with a diode array detector (DAD), a degasser, two eluent pumps, a column oven and an autosampler. The separation was performed on a reversed-phase Waters Nova-Pak C18 column (4.9 × 250 mm, 4 µm), (Water Milford, MA, USA). The mobile phase consisted of 1% aqueous formic acid (solvent A) and 1% formic acid in acetonitrile/methanol (25/75) (solvent B). Separation was obtained using the following elution gradient: 2% B isocratic for 10 min, from 2% to 98% B linear for 30 min, 98% B isocratic for 7 min. The flow rate was 0.6 mL/min, and the injection volume was 10 µL. The column oven was set at 30 °C. The mass spectral data were acquired with the following ESI inlet conditions: nebulising gas and drying gas were nitrogen at a flow rate of 3.0 and 15.0 L/min, respectively; the interface voltage was set to −3.5 kV; desolvation line (DL) temperature was 250 °C and the heat block temperature was 400 °C. The mass spectrometer operated in Negative Ion Scan and in Product Ion Scan mode using analyte-specific precursor ions, with Argon as CID (Collision Induced Dissociation) gas at a pressure of 230 kPa. Quantification of the single phenolic compounds was directly performed by HPLC–DAD in triplicates. In particular, six individual compounds, i.e., gallic acid, epicatechin, myricetin 3-*O*-rhamnoside, quercetin 3-*O*-rhamnoside, rutine, were quantified with their own standard curves. Calibration of epicatechin, myricetin and kaempferol derivatives was performed at 280 and 350 nm using epicatechin, myricetin 3-*O*-rhamnoside and kaempferol 7-*O*-glucoside as reference compounds, respectively.

### 3.4. Superoxide Scavenging Activity

The scavenging activity of sample extracts on superoxide was measured according to a modified version of the method reported by Nishikimi, Rao and Yagi [52]. Superoxide anion was generated enzymatically by xanthine/xanthine oxidase system. Sample extracts were added in the concentration range of 1.95–40 µM to the reaction mixture consisting of xanthine 0.3 mM and 0.3 mM NBT dissolved in potassium phosphate buffer (pH 7.4) with 0.05 mM EDTA (PBE). Finally, 1 mL of xanthine oxidase (0.09 units/mL PBE) was added to the mixture and incubated at 37 °C for 20 min. The absorbance of NBT was measured at 560 nm. The superoxide scavenging activity was expressed as percent (%) superoxide quenching, which was calculated as (1 − B/A) × 100, where B and A are the activities of xanthine oxidase with and without the addition of sample extracts, respectively.

### 3.5. Hydroxyl Radical-Scavenging Activity

The scavenging activity of sample extracts on hydroxyl radicals was measured according to the method of Smirnoff and Cumbes [53]. The reaction mixture consisted of FeSO_4_ (1.5 mM), hydrogen peroxide (6 mM), sodium salicylate (20 mM) and various concentrations of extracts (0.065–13 µM). The reaction mixture was incubated at 37 °C for 1 h in a water bath. After incubation the absence of the hydroxylated salicylate complex was measured spectrophotometrically at 562 nm. The percentage of hydroxyl radical scavenging activity was calculated by the following formula: % scavenging activity = [1 − (A_1_ − A_2_)/A_0_] × 100, where A_0_ was absorbance of the control without extracts, A_1_ was the absorbance in the presence of the extract, and A_2_ was the absorbance without sodium salicylate.

### 3.6. DPPH Radical-Scavenging Activity

The extracts were tested for in vitro DPPH Radical-Scavenging activity following the protocol described by Baratto et al. [54] with some modifications. The EPR (Electron Paramagnetic Resonance) signal of the DPPH radical was monitored before and after the addition of extracts and standards. Measurements were performed on a X-band (ν = 9 GHz) Bruker Elexsys E500 Series spectrometer (BRUKER DALTONIK GmbH, Germany) with an ER4122SHQE cavity. Spectra were recorded using the following experimental conditions: temperature 298 K, microwave frequency 9.865 GHz, central field 351.7 mT, scan width 10 mT, microwave power 4 mW, modulation frequency 100 kHz, modulation amplitude 0.1 mT. 0.1 mL of 0.2 mM ethanol solution of DPPH were mixed with 0.1 mL of ethanol (blank) or with an equal volume of each extract, in the concentration range of 0.065–13 µM. The obtained mixture was shaken and left at room temperature for 20 min. To determine the scavenging capacity, the area of the EPR radical signal was calculated through a double integral of the experimental spectrum. DPPH scavenging capacity was obtained by the following equation: % scavenger = (1 − A/A0) × 100 where A is the area of the DPPH signal in the presence of extract or standard and A0 is the area of the DPPH signal alone. IC_50_ values were calculated and compared with standards of myricitrin and epicatechin.

### 3.7. Statistical Analysis

All the experiments were conducted in triplicates, and the data were presented as mean ± SD (standard deviation). SPSS (version 23; SPSS Inc., Chicago, IL, USA) was used to process the results. For the DPPH assay a one-way ANOVA test followed by Tukey’s test (*p* < 0.05) was used to analyze the differences among IC_50_ of the CEE and its various fractions.

## 4. Conclusions

The purpose of this study was to investigate the polyphenolic composition of a crude ethanolic leaf extract of *C. incanus*. We focused on obtaining three different polyphenolic enriched fractions in an attempt to make systematic comparisons among their antioxidant activities and to identify the major antioxidative components of *C. incanus* leaves. Among all the fractions analysed, the ethyl acetate fraction was found to be the most effective in terms of radical scavenging activity. These results offer clear evidence that the flavonol enriched fraction obtained from *C. incanus* leaves could be a suitable target for further in vivo antioxidant studies.

## Figures and Tables

**Table 1:** HPLC–DAD-MS/MS characterisation of main polyphenols present in crude ethanolic leaf extract (CEE) of *C. incanus*. Compounds numbers correspond to those indicated in Figure 1. (n.d *, not detected; sh, shoulder).

Peak *n*	t_R_ (min)	Λ (nm)	[M-H]^−^ (m/z)	MS^2^ (m/z)	Tentative Assignement
1	4.6	234,270	331	125, 169	Monogalloyl glucose
2	8.3	234,272	169	125	Gallic acid
3	16.9	236,272	609	441, 423, 483, 305, 303	(Epi)Gallocatechin dimer
4, 5	19.5	234,272	305	611, 125, 137	(−)-Gallocatechin and (−)-epigallocatechin
6	20.6	236,276	593	407, 467, 425, 289, 285	(Epi)gallocatechin-(epi)catechin or (Epi)catechin-(epi)gallocatechin
7, 8	21.5	236,278	289	245, 205	(+) Catechin and (−) Epicatechin
9	24.2	260,360	n.d *	-	Myricetin derivative 1
10	24.5	254,362	479	316, 271	Myricetin-3-*O*-hexoside
11	25.4	260,360	n.d *	-	Myricetin derivative 2
12	25.6	260,358	463	316, 271, 179	Myricitrin
13	25.7	256,356	609	301	Rutin
14	26.6	265,355	433	301, 271	Quercetin-3-*O*-pentoside
15	26.9	256,350	447	301, 179	Quercitrin
16	27.8	264,352	n.d *	-	Quercetin derivative 1
17	28.2	264,352	n.d *	-	Quercetin derivative 2
18	29.5	264,314,346sh	593	285, 145	Kaempferol 3-*O*-rutinoside
19	33.3	268,314,348sh	739	285, 306, 145, 452	Kaempferol-3-(3″,6″-dicoumaroyl)-glucose

**Table 2:** Mean concentration of phenylpropanoids (μmol/mL) in CEE and enriched fractions of *Cistus incanus* leaves (*n* = 3).

Sample	Monogalloyl Glucose and Gallic Acid	Catechins Derivatives ^a^	Myricetin Derivatives ^b^	Quercetin Derivatives ^c^	Kaempferol Derivatives ^d^	Proanthocyanidin Polymers
CEE	0.315 ± 0.024	2.256 ± 0.076	2.719 ± 0.148	3.578 ± 0.217	0.055 ± 0.009	55.376 ± 3.067
EAF	0.236 ± 0.019	1.647 ± 0.069	2.202 ± 0.127	3.140 ± 0.162	0.036 ± 0.004	nd
AF1	nd	nd	nd	nd	nd	25.193 ± 0.597
AF2	nd	nd	nd	nd	nd	31.037 ± 0.901

nd = not detectable. ^a^ (Epi)gallocatechin dimer, (−)-Gallocatechin, (−)-Epigallocatechin, (Epi)gallocatechin-(epi)catechin, (+)-Catechin, (−)-Epicatechin; ^b^ Myricetin derivative 1, Myricetin-3-*O*-hexoside, Myricetin derivative 2, Myricitrin; **^c^** Quercetin-3-*O*-pentoside, Quercitrin, Quercetin derivative 1, Quercetin derivative 2; ^d^ Kaempefol-3-*O*-rutinoside, Kaempferol-3-(3″,6″-dicoumaroyl)-glucose. EAF = Ethyl acetate Fraction, AF1 = Aqueous Fraction 1, AF2 = Aqueous Fraction 2.

**Table 3:** IC_50_ (half maximal inhibitory concentration, μM) of different extracts and standards in superoxide anion, hydroxyl and DPPH (1,1-diphenyl-2-picrylhydrazyl) radical scavenging assays. Each value in the table is represented as Mean ± SD (*n* = 3). Means not sharing the same letter are significantly different at *p* < 0.05 probability level in each column. CEE: Crude Ethanolic Extract; EAF = Ethyl acetate Fraction, AF1 = Aqueous Fraction 1, AF2 = Aqueous Fraction 2, MYR = Myricitrin Standard, EPI = Epicatechin Standard.

Sample	IC_50_ (μM)		
	Superoxide Anion Radical	Hydroxyl Radical	DPPH Radical
CEE	20.47 ± 1.05 ^b^	0.68 ± 0.05 ^c^	2.99 ± 1.18 ^b^
EAF	5.47 ± 0.98 ^d^	0.52 ± 0.05 ^d^	0.92 ± 0.10 ^c^
AF1	24.99 ± 2.10 ^a^	0.99 ± 0.08 ^a^	11.78 ± 0.85 ^a^
AF2	22.80 ± 1.19 ^a^	1.09 ± 0.12 ^a^	10.92 ± 0.38 ^a^
MYR	4.86 ± 0.86 ^d^	0.44 ± 0.03 ^d^	0.68 ± 0.07 ^c^
EPI	12.20 ± 1.65 ^c^	0.83 ± 0.07 ^b^	1.49 ± 0.27 ^b,c^

## Supplementary Materials

Supplementary materials can be found at www.mdpi.com/1422-0067/17/8/1344/s1.
