# Supplementary material for: Characterisation and Antioxidant Activity of Crude Extract and Polyphenolic Rich Fractions from C. incanus Leaves
Source: Int J Mol Sci. 2016 Aug 17;17(8):1344. doi: 10.3390/ijms17081344 (PMC5000740; doi:10.3390/ijms17081344)
Supplement: Supplementary file 1 [file ijms-17-01344-s001.pdf]

Supplementary Material: Characterisation and Antioxidant Activity of Crude Extract and Polyphenolic Rich Fractions from *C. incanus* Leaves

Antonella Gori, Francesco Ferrini, Maria Cristina Marzano, Massimiliano Tattini, Mauro Centritto, Maria Camilla Baratto, Rebecca Pogni and Cecilia Brunetti

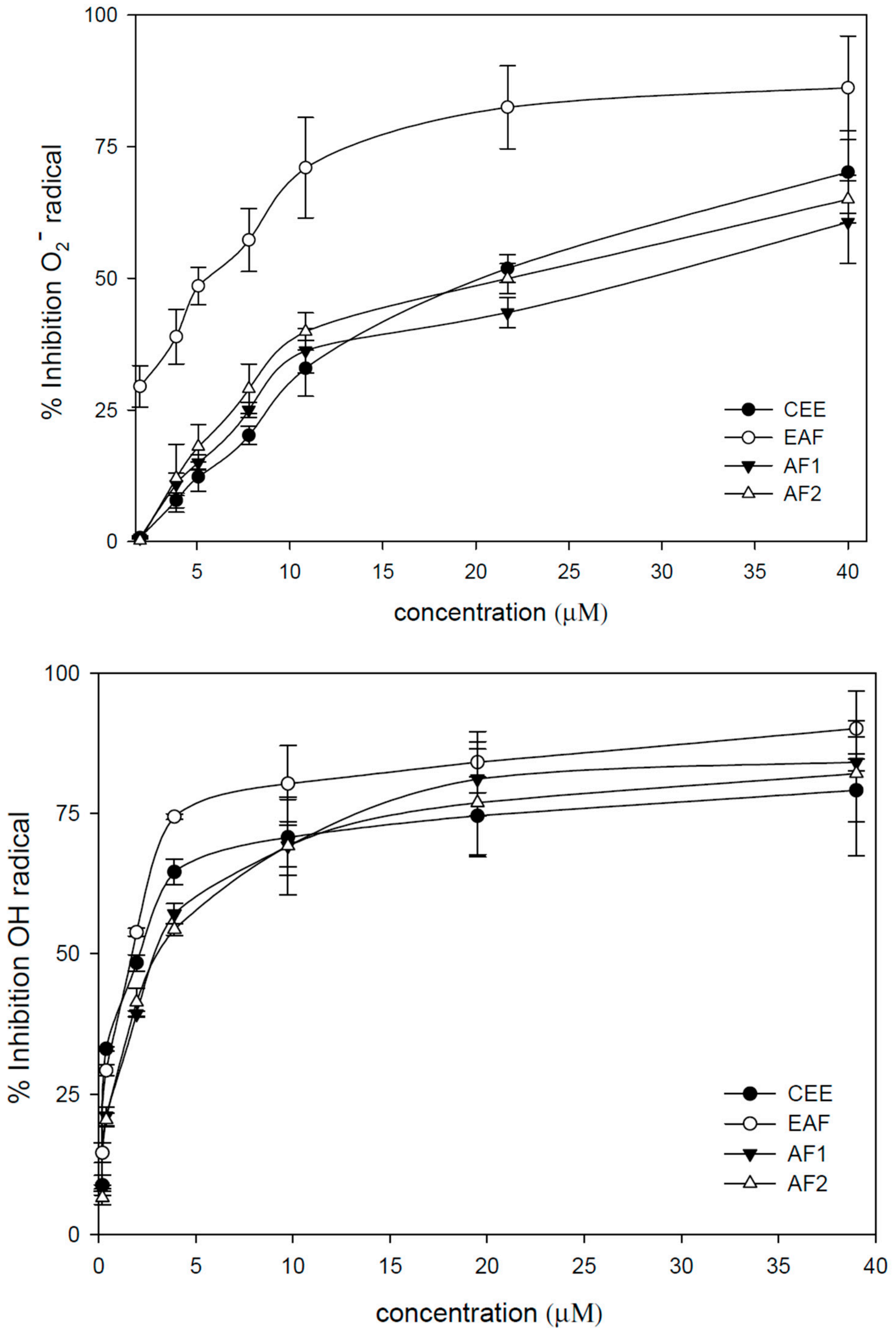

Figure S1. *Cont.*

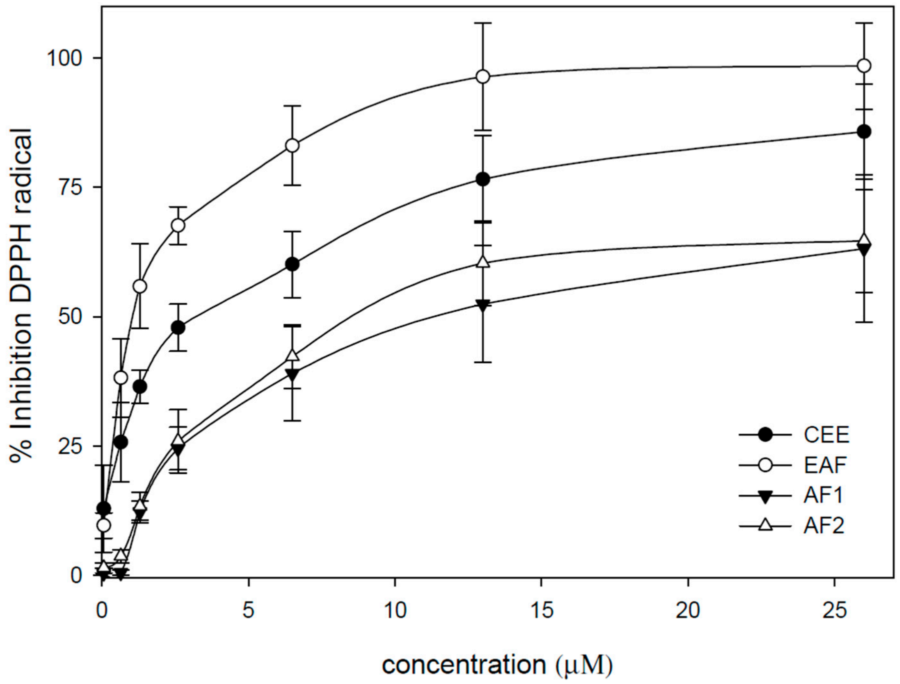

Figure S1. Scavenging activity (% of Inhibition) of different concentrations of CEE (crude ethanolic extract), EAF (ethyl acetate fraction), AF1 (aqueous fraction 1) and AF2 (aqueous fraction 2) of *C. incanus* leaves on superoxide anion (O_2^-), hydroxyl (OH) and DPPH radicals. Values are the average of triplicate experiments and represented as Mean ± Standard Deviation. DPPH, 1,1-diphenyl-2-picrylhydrazyl.
